# Biliary Phospholipids Sustain Enterocyte Proliferation and Intestinal Tumor Progression via Nuclear Receptor Lrh1 in mice

**DOI:** 10.1038/srep39278

**Published:** 2016-12-20

**Authors:** Michele Petruzzelli, Elena Piccinin, Claudio Pinto, Claudia Peres, Elena Bellafante, Antonio Moschetta

**Affiliations:** 1 Department of Interdisciplinary Medicine, “Aldo Moro” University of Bari, 70124 Bari, Italy; 2 Fondazione Mario Negri Sud, Santa Maria Imbaro, 66030 Chieti, Italy; 3 National Cancer Research Center, IRCCS Istituto Oncologico “Giovanni Paolo II”, 70124 Bari, Italy; 4 INBB, National Institute for Biostuctures and Biosystems, 00136 Rome, Italy

## Abstract

The proliferative-crypt compartment of the intestinal epithelium is enriched in phospholipids and accumulation of phospholipids has been described in colorectal tumors. Here we hypothesize that biliary phospholipid flow could directly contribute to the proliferative power of normal and dysplastic enterocytes. We used *Abcb4*^−/−^ mice which lack biliary phospholipid secretion. We first show that *Abcb4*^−/−^ mice are protected against intestinal tumorigenesis. At the molecular level, the transcriptional activity of the nuclear receptor Liver Receptor Homolog-1 (Lrh1) is reduced in *Abcb4*^−/−^ mice and its re-activation re-establishes a tumor burden comparable to control mice. Feeding *Abcb4*^−/−^ mice a diet supplemented with phospholipids completely overcomes the intestinal tumor protective phenotype, thus corroborating the hypothesis that the absence of biliary phospholipids and not lack of *Abcb4* gene *per se* is responsible for the protection. In turn, phospholipids cannot re-establish intestinal tumorigenesis in *Abcb4*^−/−^ mice crossed with mice with intestinal specific ablation of *Lrh1*, a nuclear hormone receptor that is activates by phospholipids. Our data identify the key role of biliary phospholipids in sustaining intestinal mucosa proliferation and tumor progression through the activation of nuclear receptor *Lrh1*.

The physiology of the intestinal mucosa is characterized by continuous cycles of epithelial proliferation, which is guaranteed by specific compartmentalization of cells into crypt-villus units. Cells at the bottom of the crypt display an activated *WNT* pathway, which leads to the nuclear translocation of β-catenin, and the transcription of a set of genes involved in cell proliferation; on the contrary, under physiological conditions, intestinal cells outside the bottom of the crypt lack WNT signaling, thus undergoing β-CATENIN degradation, and arrested cell proliferation^1^. Bile flow represents a critical physiologic link between the liver and intestine, responsible for synchronizing enterohepatic homeostasis during food intake and facilitating nutrient digestion, lipid absorption, and the disposal of excessive endogenous cholesterol^2^. Multiple clues suggest that intestinal phospholipids may play a role in intestinal mucosa regeneration and colon rectal cancer (CRC) pathophysiology.

The crypt compartment of the intestinal mucosa is enriched in total phospholipids, when compared to mid-villus and villus-tip regions^3^, and isolated crypt cells display increased lipid synthesis compared to upper villus cells^4^, suggesting a role for phospholipids in proliferating enterocytes. Furthermore, accumulation of phospholipids has been described in multiple forms of human cancer, including those of the colon^5^,^6^, mammary gland^7^ and uterus^8^. Also, it has been shown that genetic mutations leading to malignant transformation determine a parallel increase in intracellular phospholipid levels^9^,^10^. The causative or compensatory nature of phospholipid accumulation in cancer tissue has not yet been ascertained.

Liver receptor homolog 1 (Lrh1) is a member of the 5 A subfamily of nuclear receptor. If previous study described Lrh1 as constitutively active, recently the interaction between specific phospholipids and nuclear receptor Lrh1 has been described. Phosphatidylcholines acts as an endogenous ligand, that binds to Lrh1 in a functional hormone-binding domain^11^. High level of Lrh1 are found in tissue of endodermal origin (liver, pancreas, intestine), where it exterts a central role in several aspects of life, ranging from the regulation of the earliest events in development to the reverse cholesterol transport and bile acid metabolism in adult liver^12^,^13^,^14^. Moreover, Lrh1 is highly expressed in intestinal crypts and it is involved in the regulation of cell renewal and inflammatory response^15^.

In the present study we focus on the role of phospholipids in the intestinal epithelium pathophysiology. Intestinal phospholipids are derived both from diet and from bile, with biliary phospholipids representing the most important source of endogenous phospholipids in the intestine^16^. We used *Abcb4*^−/−^ mice^17^, which lack the hepatic transporter responsible for the flippase and active secretion of phospholipids across the hepatocyte canalicular membrane into bile. These mice have absence of biliary derived intestinal phospholipids with lower intraluminal phospholipid content. We then show that the absence of intestinal phospholipids promote a strong reduction of intestinal cell proliferation, with a significant decreases in mucosal length. Concomitantly, we observe that Lrh1 activation due to the presence of phospholipids leads to a significant increase in tumor number and size in two distinct models of intestinal carcinogenesis. The specific intestinal ablation of Lrh1 reverse this phenotype, even in presence of dietary phospholipids. Therefore, intestinal phospholipid accumulation promotes enterocyte regeneration and tumor progression.

## Results

### Lrh1 activation in Abcb4^−/−^ mice

**Figure 1:**
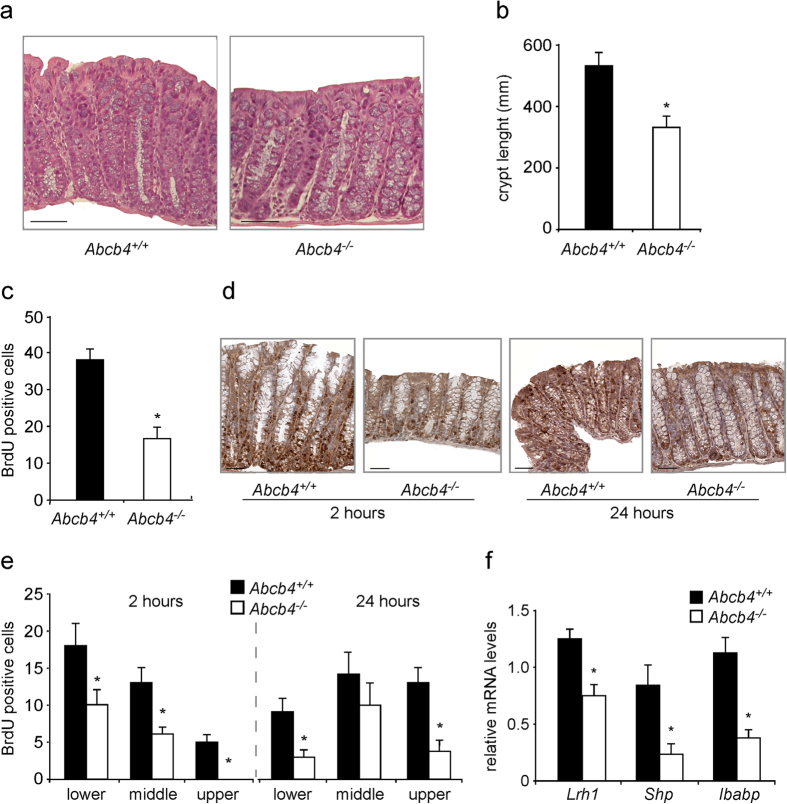
Reduced colonic crypt length and decreased cell proliferation in the absence of biliary derived intraluminal phospholipids. (**a**) Hematoxylin and eosin (H&E) staining of colon sections from *Abcb4*^+/+^ and *Abcb4*^−/−^ mice (magnification 200x). Note the reduced crypt length in *Abcb4*^−/−^ mice. (**b**) Quantification of crypt length by morphometric analysis. (**c**) Proliferation of enterocytes in *Abcb4*^+/+^ and *Abcb4*^−/−^ mice analyzed by BrdU staining. Quantification of total BrdU-positive cells showing reduced colonic enterocyte proliferation in *Abcb4*^−/−^ mice. *Significantly different from *Abcb4*^+/+^ mice (*p *< 0.05). (**d**) Representative images of colon sections at 2 and 24 hrs after BrdU injection are shown (*n *= 5 mice per group). (**e**) Number of BrdU positive cells per segment in the crypt to epithelium axis at 2 and 24 hrs. after BrdU injection in *Abcb4*^+/+^ mice (black bars) and *Abcb4*^−/−^ mice (white bars). At 2 hrs. BrdU staining was predominant but not confined to the lower zone of the crypts in *Abcb4*^+/+^ mice, while in *Abcb4*^−/−^ mice BrdU-positive cells were located exclusively in the bottom two-thirds of crypts. At 24 hrs. very few BrdU-positive cells reached the top third of the epithelium in *Abcb4*^−/−^ mice, thus indicating defective cell migration. (**f**) mRNA expression in colon samples of *Abcb4*^+/+^ mice (black bars) and *Abcb4*^−/−^ mice (white bars), quantified by qRT-PCR. Gene expression analysis revealed reduced mRNA levels of nuclear receptor *Lrh1* and its target genes *Shp* and *Ibabp* in *Abcb4*^−/−^ mice.

**Figure 2:**
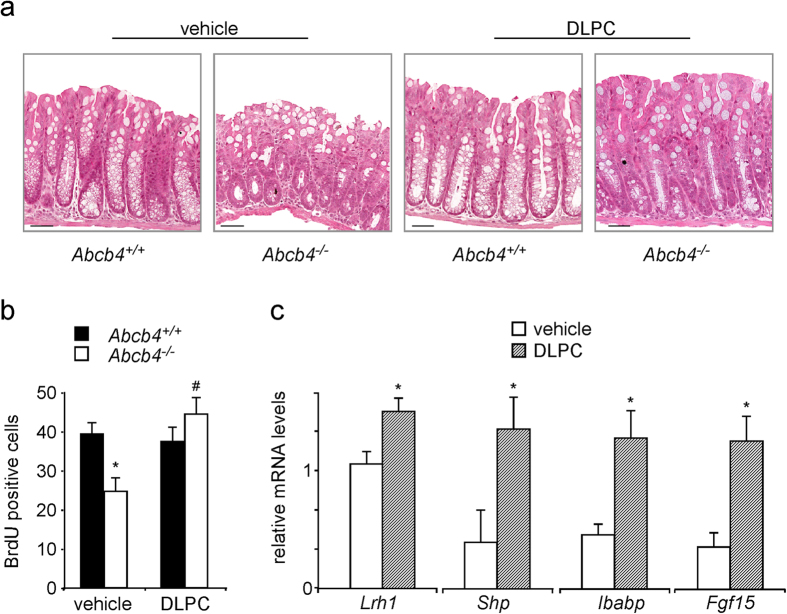
Reduced ileal crypt length and decreased cell proliferation in Abcb4^−/−^ mice are rescued by treatment with Lrh1 agonist DLPC. (**a**) Hematoxylin and eosin (H&E) staining of colon sections from *Abcb4*^+/+^ and *Abcb4*^*−*/*−*^ mice treated with vehicle or DLPC (magnification 200x). (**b**) Quantification of BrdU-positive cells showing a reduction of positive cells in ileum of *Abcb4*^−/−^ mice (white bars), which was restored to levels comparable to *Abcb4*^+/+^ mice (black bars) after DLPC-treatment. (**c**) mRNA expression in ileum samples from *Abcb4*^−/−^ mice treated with vehicle or DLPC showing increased levels of *Lrh1, Shp, Ibabp*, and *Fgf15*. All data are represented as mean ± SEM. *Significantly different from all other conditions (*p* < 0.05); significantly different DLPC versus vehicle (*p* < 0.05).

In the present study, we examined the role of biliary phospholipids in intestinal tumorigenesis and mucosa regeneration exploiting *Abcb4*^−/−^ mice (Abcb4, ATP Binding Cassette Subfamily B Member 4)^17^. Colon from *Abcb4*^−/−^ mice, displayed a striking reduction in crypt length and cell proliferation, when compared to littermate controls (Fig. 1a–d). Furthermore, cell migration was delayed in *Abcb4*^−/−^ mice (Fig. 1c–e). To dissect the molecular determinants of the observed phenotypes, we measured expression levels of candidate genes. Liver Receptor Homolog 1 (LRH1) is a nuclear receptor highly expressed in the intestinal crypts^18^, where it promotes mucosa regeneration and initiation of intestinal tumorigenesis^15^,^19^. In line with the recently reported activity of phospholipids as endogenous ligands for Lrh1^20^,^21^,^22^, absence of biliary phospholipids in *Abcb4*^−/−^ mice resulted in nearly halved mRNA levels of *Lrh1*, together with a severe relative fold reduction in expression levels of its target genes Small Heterodimer Partner (*Shp*) and Intestinal Bile Acid Binding Protein (*Ibabp*) (Fig. 1f). We next treated mice with the 1,2-dilauroyl-sn-glycero-3-phosphocholine (DLPC)^11^,^23^, to ascertain whether reactivation of Lrh1 would rescue intestinal mucosa proliferation in *Abcb4*^−/−^ mice. DLPC is an Lrh1 agonist, which binds directly to Lrh1 and promotes its activation^11^,^23^. Ten days of DLPC treatment restored crypt length and cell proliferation rate in *Abcb4*^−/−^ mice to levels comparable to littermate controls (Fig. 2a,b), along with increased expression of *Lrh1* and its target genes *Shp, Ibabp*, and *Fgf15* (Fibroblast Growth Factor 15) (Fig. 2c), thereby supporting a causative role for *Lrh1* in the reduced proliferative phenotype displayed by *Abcb4*^−/−^ mice.

### Intestinal tumorigenesis in Abcb4^−/−^ mice

**Figure 3:**
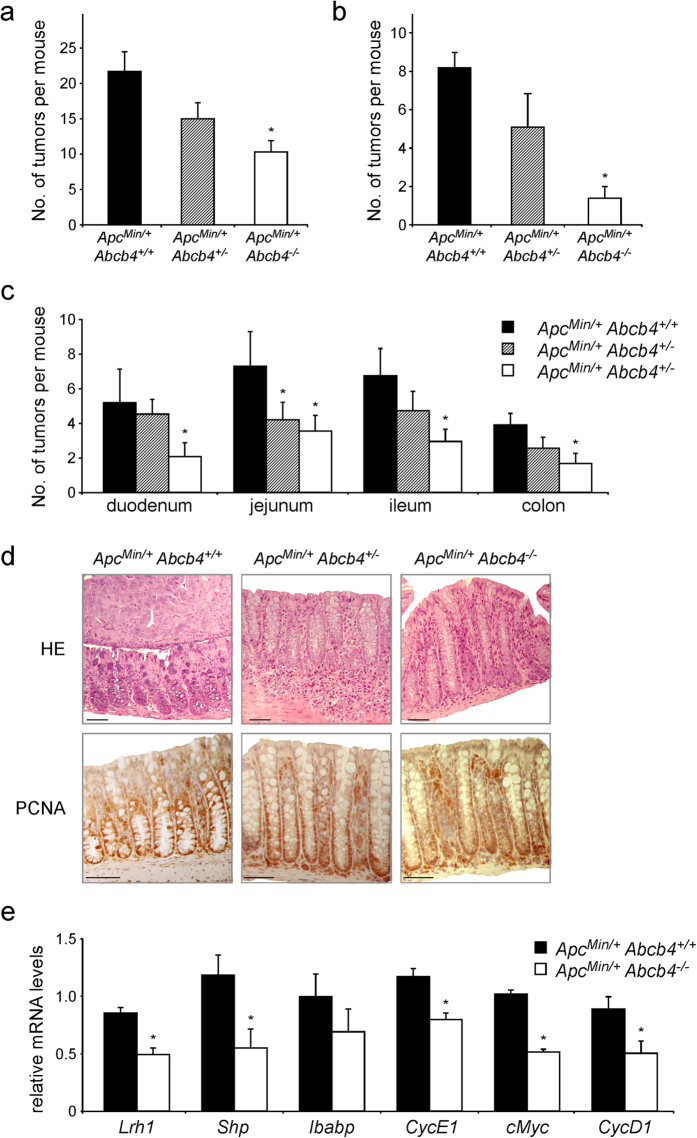
Absence of biliary derived intraluminal phospholipids protects Abcb4^−/−^ mice from intestinal tumorigenesis in the genetic model of ApcMin^/+^ mice. *Apc*^*Min*/+^*/Abcb4*^+/+^ mice (n = 12), heterozygous *Apc*^*Min*/+^*/Abcb4*^+/−^ (n = 12), and homozygous *Apc*^*Min*/+^*/Abcb4*^−/−^ mice (n = 13) were sacrificed at six months of age and intestinal tumor formation was analyzed. Compared to *Apc*^*Min*/+^*/Abcb4*^+/+^ mice (black bars), average number of tumors per mouse (**a**) and average number of tumors above 2 mm of diameter (**b**) were reduced in *Apc*^*Min*/+^*/Abcb4*^+/−^ (gray bars), and even further in *Apc*^*Min*/+^*/Abcb4*^−/−^ (white bars). (**c**) Average number of tumors per mouse per intestinal district. (**d**) H&E and immunohistochemical detection of proliferative marker Pcna in *Apc*^*Min*/+^/*Abcb4*^+/+^ *Apc*^*Min*/+^/*Abcb4*^+/−^ and *Apc*^*Min*/+^/*Abcb4*^*−*/*−*^ mice. *Apc*^*Min*/+^/*Abcb4*^+/+^ mice displayed adenomatous colon, with an expansion of the basal proliferative compartment (Pcna staining). *Apc*^*Min*/+^/*Abcb4*^+/+^ exhibited altered colon morphology. On the contrary, specimens from *Apc*^*Min*/+^/*Abcb4*^*−*/*−*^ revealed a less altered colon morphology together with a decreased Pcna labeling. (**e**) Gene expression analysis from colon samples of *Apc*^*Min*/+^*/Abcb4*^+/+^ mice (n = 12, black bars) and *Apc*^*Min*/+^*/Abcb4*^−/−^ mice (n = 13, white bars) showed reduced mRNA levels of nuclear receptor *Lrh1* and its target genes *Shp, Ibabp*, and *CyclinE1*, and ß-catenin/Tcf4 targets *cMyc* and *CyclinD1*, in *Abcb4*^−/−^ mice. (**f**) Immunoblots were performed with specific antibodies against PCNA, CyclinD1 and β-Actin on colon samples isolated from *Apc*^*Min*/+^/*Abcb4*^+/+^*, Apc*^*Min*/+^/*Abcb4*^+/−^ *and Apc*^*Min*/+^/*Abcb4*^*−*/*−*^. Protein level of PCNA and CyclinD1 were quantified and normalized against β-Actin. All data are represented as mean ± SEM. *Significantly different from *Apc*^*Min*/+^*/Abcb4*^+/+^ mice (*p* < 0.05).

**Figure 4:**
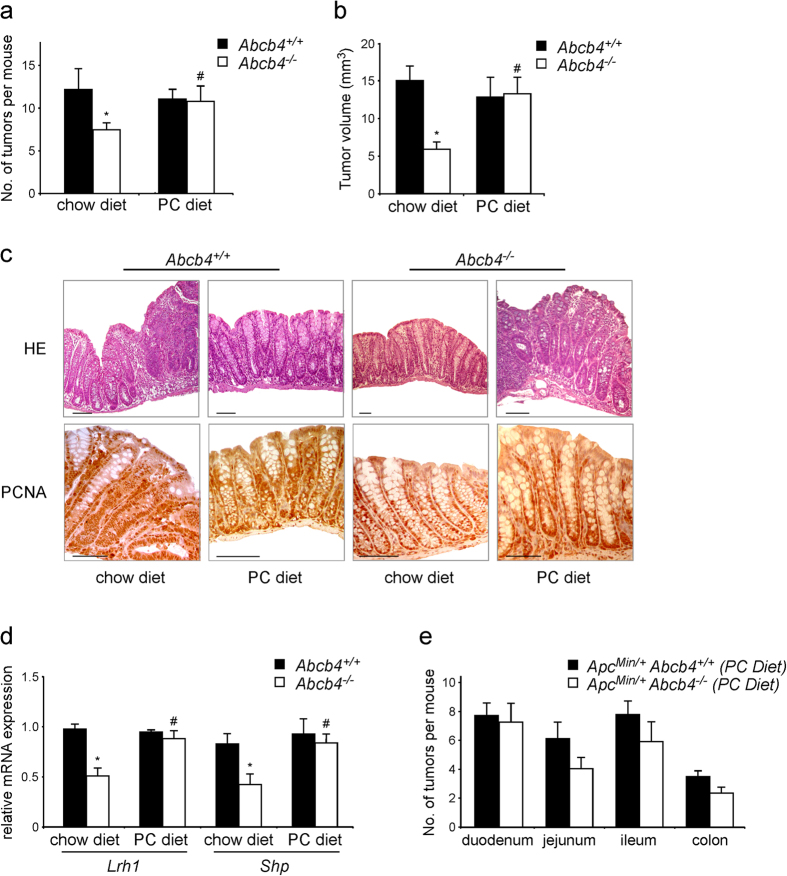
Dietary phospholipids abolish the tumor protective phenotype of Abcb4^−/−^ in the chemical induced colitis carcinogenesis model and in the genetic model of ApcMin^/+^ mice. *Abcb4*^+/+^ mice (n = 10) and *Abcb4*^−/−^ mice (n = 12) were s Average number of tumors per mouse (**a**) and average tumor volume (**b**) of macroscopic tumors observed in colon of *Abcb4*^+/+^ mice (black bars) and *Abcb4*^−/−^ mice (white bars) mice subjected to AOM and DSS treatment, in conditions of chow diet and diet supplemented with phospholipids. Tumor number and volume were drastically reduced in *Abcb4*^−/−^ mice. (**c**) H&E staining and immunohistochemical analysis of colon specimens in the chemical induced colitis carcinogenesis model. Enhanced epithelial proliferation in *Abcb4*^+/+^ mice was evident as expansion of the PCNA positive compartment. In contrast, colon specimens from *Abcb4*^*−*/*−*^ mice displayed decreased PCNA labeling. Labeling for PCNA was increased to levels similar to those of *Abcb4*^+/+^ mice after the PC diet. (**d**) Gene expression analysis from colon specimens of AOM-DSS treated *Abcb4*^+/+^ mice (n = 10, black bars) and *Abcb4*^−/−^ mice (n = 12, white bars) showing halved mRNA levels of nuclear receptor *Lrh1* and its target gene *Shp* in *Abcb4*^−/−^ mice; notably, expression levels of both *Lrh1* and *Shp* were restored to control values after the PC diet. In (**e**) is represented average number of tumors per mouse per intestinal district for *Apc*^*Min*/+^*/Abcb4*^+/+^ mice (n = 10) and *Apc*^*Min*/+^*/Abcb4*^−/−^ mice (n = 10) fed with diet supplemented with phospholipids. Feeding mice a diet enriched in PC diet abrogated the difference in tumor multiplicity and volume between *Abcb4*^+/+^ and *Abcb4*^−/−^ mice in both chemical tumor model and genetic one. All data are represented as mean ± SEM. *Significantly different from all other conditions (*p* < 0.05); ^#^significantly different PC diet versus chow diet (*p* < 0.05).

The effect of absent biliary phospholipids on intestinal tumor formation was investigated in *Apc*^*Min*/+^ (Apc, Adenomatous Polyposis Coli) mice, a genetic model of intestinal tumors^24^,^25^. In line with the reduced epithelial proliferation observed in the intestine of *Abcb4*^−/−^ mice, *Apc*^*Min*/+^/*Abcb4*^−/−^ mice displayed protection against intestinal tumor formation, when compared to *Apc*^*Min*/+^/*Abcb4*^+/+^ (Fig. 3a–d). Interestingly, heterozygosis for *Abcb4* gene in *Apc*^*Min*/+^/*Abcb4*^+/−^ mice resulted in a tumor phenotype intermediate between *Apc*^*Min*/+^/*Abcb4*^+/+^ and *Apc*^*Min*/+^/*Abcb4*^−/−^ mice (Fig. 3a–d). Biliary phospholipid levels are halved in *Abcb4*^+/−^ mice^17^, thus suggesting a concentration-dependent role of phospholipids in quantitative progression of intestinal cancer. Transcriptional analysis confirmed the reduction in mRNA levels of *Lrh1* and its target genes *Shp* and *Ibabp* in *Apc*^*Min*/+^/*Abcb4*^−/−^ mice compared to *Apc*^*Min*/+^/*Abcb4*^+/+^ mice (Fig. 3e). Of note, we observed also a reduction in the transcriptional levels of *CyclinE1* (Fig. 3e), yet another direct *Lrh1* target gene, with a critical role in cell cycle progression and cell proliferation^19^. Moreover, protein analysis revealed a downregulation of CyclinD1 and PCNA (Proliferating Cell Nuclear Antigen), both markers of cell proliferation (Supplementary Fig. 1A,B). Lastly, expression levels of β-catenin-Transcription Factor 4 (TCF4) targets, proto-oncogenes *cMyc* and *CyclinD1*, were also significantly reduced (Fig. 3e), in line with the documented role of *Lrh1* as co-activator for β-catenin-TCF4 transcriptional complex^19^. To further confirm the protection displayed by *Abcb4*^−/−^ mice against intestinal tumorigenesis, we challenged mice with a complementary experimental setting, the azoxymethane-Dextran sulfate sodium (AOM/DSS) colitis-carcinogenesis model. Compared to littermate controls, *Abcb4*^−/−^ mice challenged with AOM/DSS developed fewer and smaller colonic tumors (Fig. 4a–c). The *Abcb4* gene is not expressed in the intestine^26^,^27^. Nevertheless, to rule out the possibility that the lack of *Abcb4* gene *per se*, rather than the absence of biliary phospholipids, was responsible for the protection against intestinal tumors, mice were fed a diet supplemented with phospholipids (PC diet)^28^. *Abcb4*^−/−^ mice fed the PC diet lost the protection against AOM/DSS, displaying colon tumor number and size similar to that of *Abcb4*^+/+^ mice (Fig. 4a,b). At the molecular level, expression of *Lrh1* and its target gene *Shp* was restored to control values after the PC diet (Fig. 4d). No difference in expression levels of the prostaglandin synthesis enzyme *Cyclooxygenase2* (Cox2) was found between *Abcb4*^+/+^ and *Abcb4*^−/−^ mice (data not shown), suggesting that reduction of the inflammatory process is not the mechanism responsible for the protection against colon cancer observed in *Abcb4*^−/−^ mice. In fact, mRNA levels of *Tumor Necrosis Factor-α (Tnfα)* and *Interleukin-1β* (data not shown) were even increased in the colon mucosa of *Abcb4*^−/−^ mice, further ruling out the hypothesis that reduced inflammation can be the mechanism of protection in the colitis carcinogenesis model. Remarkably, the intestinal phenotype observed in AOM/DSS treated *Abcb4*^*−*/*−*^ mice, namely decreased tumorigenesis and increased inflammatory response, is very similar to that seen in mice with haploinsufficiency of *Lrh1*^15^,^29^. Also in *Apc*^*Min*/+^/*Abcb4*^−/−^, the introduction of dietary PC was able to restore the intestinal tumor number very close to the one of *Apc*^*Min*/+^/*Abcb4*^+/+^ (Fig. 4e). Thus, both the genetically induced and carcinogen-induced models of intestinal tumors show that *Abcb4*^−/−^ mice are protected against intestinal carcinogenesis, and that a diet supplemented with PC completely overcomes this protection.

### Intestinal ablation of LRH1 in Abcb4^−/−^ mice

**Figure 5:**
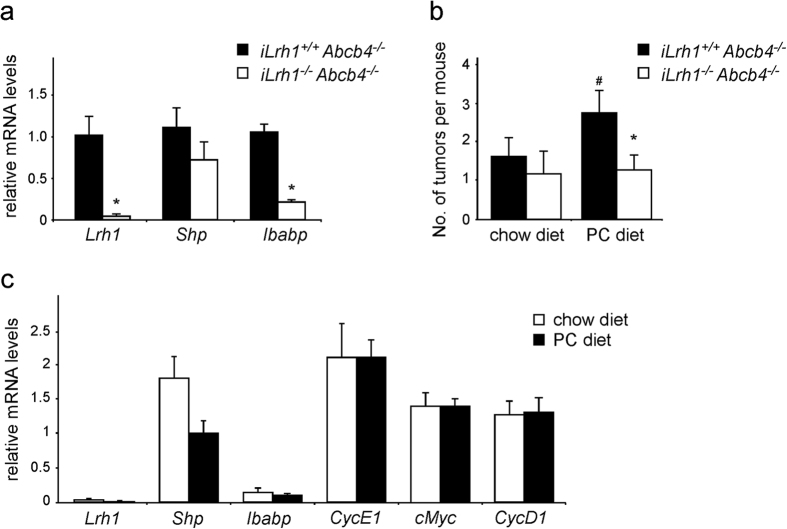
Absence of intestinal Lrh1 sustains the tumor protective role of *Abcb4*^−/−^. (**a**) Gene expression analysis from colon samples of *iLrh1*^+/+^*/Abcb4*^−/−^ mice versus *iLrh1*^−/−^*/Abcb4*^−/−^ mice in basal conditions (chow diet without any treatment). (**b**) Average number of tumors per mouse observed in ileum and colon of *iLrh1*^+/+^*/Abcb4*^−/−^ mice (black bars) and *iLrh1*^−/−^*/Abcb4*^−/−^ mice (white bars) subjected to AOM and DSS treatment and fed with chow diet and diet supplemented with phospholipid. (**c**) Gene expression analysis from colon samples of *iLrh1*^−/−^*/Abcb4*^−/−^ mice fed with chow diet (white bars) and PC diet (grey bars). All data are represented as mean ± SEM. *Significantly different from *iLrh1*^+/+^*/Abcb4*^−/−^ mice (*p* < 0.05); ^#^significantly different PC diet versus chow diet (*p* < 0.05).

To better clarify the role of *Lrh1* in mediating the effects of phospholipids in tumor promotion, we sought to investigate the absence of intestinal *Lrh1 (iLrh1*^−/−^) in *Abcb4*^−/−^ mice. Gene expression profile of colon samples from *iLrh1*^−/−^/*Abcb4*^−/−^ mice confirmed *Lrh1* deletion and a substantial reduction in expression levels of Lrh1 target genes (Fig. 5a). *iLrh1*^−/−^/*Abcb4*^−/−^ mice fed with a diet supplemented with PC, were subjected to AOM/DSS treatment. Interestingly, dietary phospholipids were not able to increase intestinal tumor formation in *Abcb4*^−/−^ mice in absence of intestinal Lrh1 (Fig. 5b). Moreover, the protein level of *CyclinD1*, a direct Lrh1 target and marker of cell proliferation, are downregulated in *iLrh1*^−/−^/*Abcb4*^−/−^ fed with chow diet, and the rescue of phospholipids by diet is not able to revert the low expression observed (Supplementary Fig. 1C,D). Also, in *iLrh1*^−/−^/*Abcb4*^−/−^ mice, dietary phospholipids did not affect the expression levels of *Ibabp, CylinE1*, and other genes related to β-catenin/Tcf4 complex (Fig. 5c).

## Discussion

In the present study, we show that absence of biliary phospholipids in *Abcb4*^−/−^ mice results in reduced length of intestinal crypts, decreased cell proliferation, and delayed epithelial regeneration rate. Remarkably, absence of biliary phospholipids in *Abcb4*^−/−^ mice leads to protection against intestinal tumorigenesis in both the genetically induced and carcinogen-induced models of intestinal tumors. Absence of biliary derived intestinal phospholipids in *Abcb4*^−/−^ mice was indeed associated with reduced activation of nuclear receptor Lrh1, which has been shown to recognize phospholipids as endogenous ligands^20^,^21^,^30^. Accordingly, the reduction in cell proliferation reverted upon activation of Lrh1 by administration of its agonist DLPC, together with transcriptional up-regulation of *Lrh1* target genes. Similarly, a diet enriched in phosphatidylcholine restored tumor susceptibility in *Abcb4*^−/−^ mice only in presence of a functional intestinal Lrh1 transcriptional pathway. These data establish the nuclear receptor Lrh1 as the functional link that allows phospholipids to drive enterocyte proliferation and, under susceptible circumstances, intestinal tumorigenesis. The nuclear receptor Lrh1 has recently emerged as a crucial factor in the gut. If on one hand, Lrh1 mediates corticosterone response, thus regulating anti-inflammatory response^29^, on the other is fundamental in the promotion of cell proliferation due to its ability to bind and enhance β-Catenin, supporting the transcription of the downstream targets *CyclinE, CyclinD* and *c-Myc*^15^. The overexpression of these cell cycle regulators have been shown in human intestinal cancer and clearly contributes to cancer onset in animal models^31^,^32^,^33^.

We recognize that also modifications of intestinal phospholipid content may affect intestinal tumorigenesis indirectly by means of decreased intestinal cholesterol absorption^34^,^35^. A diet with high levels of cholesterol and saturated fats has been associated with an increased incidence of colon cancer, both in epidemiological studies^36^,^37^ and experimental models^38^, where cholesterol has been shown to act as a strong dietary co-carcinogen^39^,^40^. Intestinal cholesterol absorption is halved and fecal neutral sterol secretion is increased four-fold in *Abcb4*^−/−^ mice^41^. Therefore, it is also possible that the protection displayed by *Abcb4*^−/−^ mice against intestinal tumorigenesis may be related to modifications of intestinal cholesterol metabolism by altered biliary phospholipid composition. Another possibility is that biliary phospholipid may provide the intestinal mucosa with pro-tumor phospholipid metabolites or precursors, such as lysophosphatidic acid, which has been recently shown to increase tumor incidence in *Apc*^*Min*/+^ mice^42^. However, given the central role of intestinal Lrh1 in the observed phenotype of the present study, future studies connecting intestinal Lrh1 function with intra-enterocyte cholesterol and phospholipid metabolism are needed.

In conclusion, employing two complementary murine models of intestinal tumorigenesis, we show that biliary phospholipids sustain intestinal mucosa regeneration and tumor formation under genetic or chemical susceptibility. The reduction in tumor incidence and –even more pronounced– the reduction of tumor size or growth in *Abcb4*^−/−^ mice suggest biliary phospholipids to be critical mediators of intestinal tumorigenesis both in the early stages of tumor initiation/promotion, as well during later tumor progression. The activation of nuclear receptor Lrh1 via biliary phospholipids depicts an intriguing novel piece of the transcriptional puzzle that regulates intestinal mucosa regeneration and cancer.

## Methods

### Animal studies and procedures

Pure strain FVBN *Abcb4*^−/−^ mice were kindly provided by Albert K. Groen (Department of Paediatrics, Center for Liver, Digestive and Metabolic Diseases, University Medical Center, Groningen, The Netherlands). FVBN/*Apc*^*Min*/+^ mice were generated by crossing for more than 8 generations FVBN mice with *Apc*^*Min*/+^ mice obtained from Jackson laboratory. *Apc*^*Min*/+^/*Mdr*^+/−^ mice were generated by crossing *Apc*^*Min*/+^ mice with *Abcb4*^−/−^ mice. Then, by intercrossing *Apc*^*Min*/+^/*Abcb4*^+/−^, we generated *Apc*^*Min*/+^/*Abcb4*^−/−^ mice. Intestinal specific *Lrh1*^−/−^ mice (*iLrh1*^−/−^) were generated by crossing villin-cre mice from Jackson laboratory with floxed Lrh1 mice that were kindly provided by Drs. Kristina Schoonians and Johan Awuerx (EPFL, Lausanne, Switzerland)^14^. *iLrh1*^−/−^*/Abcb4*^−/−^ mice were generated by crossing *Abcb4*^−/−^ mice with *iLrh1*^−/−^ mice. *In vivo* Lrh1 agonism experiment was performed on 10 weeks old mice employing the natural phospholipid 1,2-dilauroyl-sn-glycero-3-phosphocholine (DLPC). DLPC (Avanti Polar Lipids) was dissolved in alcohol and administered by gavage (200 mg/Kg body weight/day in PEG400/Tween80, 4:1) once daily for ten consecutive days. Animals in the control group received vehicle only. For the genetic model of CRC formation, *Apc*^*Min*/+^, *Apc*^*Min*/+^/*Abcb4*^+/−^ and *Apc*^*Min*/+^/*Abcb4*^−/−^ were sacrificed at 6 months of age. For the chemical induced colitis carcinogenesis model^43^, pathogen free 10–16 week old male *Abcb4*^+/+^, *Abcb4*^−/−^*, iLrh1*^−/−^*/Abcb4*^−/−^ and *iLrh1*^+/+^*/Abcb4*^−/−^ mice on a pure FVBN background were injected intraperitoneally with 12 mg/Kg body weight of azoxymethane (AOM, Sigma-Aldrich, Saint Louis, MO, USA) dissolved in NaCl 0.9%. Five days later, 3% dextran sulfate sodium (DSS) was given in the drinking water over 5 days, followed by 16 days of regular water. This cycle was repeated 3 times and the body weight was measured at the end of each cycle. To evaluate the effect of a dietary phosphatidylcholine (PC) on colon cancer formation, we fed both *Abcb4*^+/+^, *Abcb4*^−/−^*, iLrh1*^−/−^*/Abcb4*^−/−^ and *iLrh1*^+/+^*/Abcb4*^−/−^ mice with a PC-enriched diet, supplemented with soybean lecithin 2.5% w/w (DP1000 mod, PC-supplemented diet, Altromin-Rieper, Vandoies, BZ, Italy) during the entire duration of AOM/DSS protocol. A minimum of eight mice per group was employed in the different experimental settings. All animals received human care according to the criteria outlined in the “Guide for the Care and Use of Laboratory Animals”. At the end of each experimental protocol, macroscopic inspection, histological analysis, and total RNA extraction were performed. Briefly, colons were removed, washed with PBS, open longitudinally and laid out. Then, they were thawed in 2.5% formalin solution at room temperature, then fixed in 70% ethanol at 4 °C for 30 min, and stained with 0.2% methylene blue for 2 min. The samples were then fixed in 10% buffered formalin, washed in 70% ethanol, and stored in this solution. Stained intestine were transferred to 2.5% formalin solution for up to 1 h and then examined in their entirety. The number of tumors was scored with a dissecting microscope by a single observer blind to the genotype of the mice or their treatment group. The results are indicated as a mean ± SEM of all the tumors counted within a single genotype group. All mice were housed under standard diet (except when indicated) provided *ad libitum* and examined daily. Genotyping was done using DNA extracted from tail biopsies of 2- to 3-week-old pups, and new breeding harems of 5 to 6 week-old mice were established to expand the population. All the experiments presented in this study have been carried out in male mice. The Ethical Committee of the Consorzio Mario Negri Sud approved this experimental set-up, which was also certified by the Italian Ministry of Health according with internationally accepted guidelines for the animal care.

### Cell proliferation assay

Intestinal epithelial cell proliferation was determined by BrdU incorporation into cell nuclei employing the 5-Bromo-2′-deoxy-uridine (BrdU). Briefly, mice were injected BrdU and sacrificed after 2 and 24 hrs. Paraffin-embedded tissue sections were stained with anti-BrdU antibody. Following immunostaining for BrdU, the number and position of BrdU-positive cells were counted from at least 30 crypts per mouse per genotype. The results are indicated as a mean ± SEM. A scheme of the crypt-to-epithelial colonic mucosal axis is reported in Supplementary Fig. 2.

### Quantitative real-time PCR (RTqPCR)

The tissues were crashed under liquid nitrogen to allow homogenization through a tissue lyser. Total RNA was isolated by Qiazol reagent (Qiagen) following the manufacture’s instruction. To avoid possible DNA contaminations, RNA was treated with DNAase (Ambion). RNA purity was also checked by spectrophotometer and RNA integrity by examination on agarose gel electrophoresis. cDNA was synthesized retro-transcribing 4 μg of total RNA in a total volume of 100 μl using High Capacity DNA Archive Kit (Applied Biosystem) and following the manufacture’s instruction. RTqPCR primers were designed using Primer Express software. The sequence of primer is listed below. PCR assays were performed in 96 well optical reaction plates using the ABI 7500HT machine (Applied Biosystems). PCR assays were conducted in duplicate wells for each sample. Baseline values of amplification plots were set automatically and threshold values were kept constant to obtain normalized cycle times and linear regression data. The following reaction mixture was used in each well: 10 μl Power Syber Green (Applied Biosystems), 2.4 μl of primers at the final concentration of 150 nM, 4.6 μl RNAase free water, 3 μl cDNA (60 ng). For all experiments the following PCR conditions were used: denaturation at 95 °C for 10 min, followed by 40 cycles at 95 °C for 15 seconds then at 60 °C for 60 seconds. Quantitative normalization of cDNA in each sample was performed using *cyclophilin* as internal control. Relative quantification was performed using the ΔΔCT method. Primer sequences are reported in Table 1.

### Histology and immunohistochemistry

Tissue specimens were fixed in 10% formalin for 12 to 24 hrs, dehydrated, and paraffin embedded. Standard immunohistochemical procedures were performed. Briefly, 5-μm-thick sections were treated with 3% hydrogen peroxide for 5 min, to quench endogenous peroxidase, and subjected to antigen retrieval by boiling the slides in antigen unmasking solution for 5 min. Sections were sequentially incubated for 60 min at room temperature in blocking protein block (Dako, Agilent Technologies, Denmark) and overnight at 4 °C with the primary antibodies (rabbit polyclonal PCNA, Santa Cruz Biotechnology, Texas, US). Sections were washed 15 min in PBS and incubated for 30 min at room temperature with the Dako Real EnVision Detecction System Peroxidase/DAB). Counterstaining was carried out with Mayer Haematoxylin (BioOptica, Italy). For negative controls, 1% nonimmune serum in PBS replaced the primary antibodies.

### Western blot analysis

Equal amounts of total tissue lysates (30 μg) were separated on a 12.5% SDS–polyacrylamide gel and transferred onto nitrocellulose membrane. Membranes were blocked with 5% BSA in TBS–0.01% Tween 20 and probed with specific antibodies against CyclinD1 (SP4, Abcam), PCNA (PC10, Santa Cruz Biotechnology) and β-Actin (Abcam) as a loading control. Membranes were finally incubated with HRPconjugated secondary antibodies and developed with a chemiluminescent reagent (Biorad). Images were acquired using Chemidoc (Biorad) and protein level were quantified using Image Lab Software (Biorad).

### Statistical analysis

All results are indicated as mean±sem. Data distribution and gene expression statistical analyses were performed using NCSS statistical and power analysis software 2007. Multiple groups were tested by one-way ANOVA repeated measures followed by Fisher’s least significant difference test for unpaired data or one way ANOVA followed by Dunns test, as appropriate. Comparisons of two groups were performed using Mann-Whitney U test. A *p* value of < 0.05 was considered significant.

## Additional Information

**How to cite this article**: Petruzzelli, M. *et al*. Biliary Phospholipids Sustain Enterocyte Proliferation and Intestinal Tumor Progression via Nuclear Receptor Lrh1 in mice. *Sci. Rep.* **6**, 39278; doi: 10.1038/srep39278 (2016).

**Publisher's note:** Springer Nature remains neutral with regard to jurisdictional claims in published maps and institutional affiliations.

## Supplementary Material

Supplementary Information

## Figures and Tables

**Table 1:** Validated primer sequences for real-time PCR.

Gene	Forward Sequence	Reverse Sequence
**Lrh1**	5′-TGGGAAGGAAGGGACAATCTT-3′	5′-CGAGACTCAGGAGGTTGTTGAA-3′
**Shp**	5′-CGATCCTCTTCAACCCAGATG-3′	5′-AGGGCTCCAAGACTTCACACA-3′
**Ibabp**	5′-TTGAGAGTGAGAAGAATTACGATGAGT-3′	5′-TTTCAATCACGTCTCCCTGGAA-3′
**CycD1**	5′-CATCCATGCGGAAAATCGT-3′	5′-TCTACGCACTTCTGCTCCTCA-3′
**CycE1**	5′-GCTTCTGCTTTGTATCATTTCTCCTC-3′	5′-GGAACCATCCATTTGACACACTT-3′
**cMyc**	5′-TGTATGTGGAGCGGTTTCTCA-3′	5′-CTGGTAGGAGGCCAGCTTCT-3′
**Fgf15**	5′-GAGCGACGGCTCTGTGGA-3′	5′-CCTTGATGGCAATCGTCTTCA-3′

## References

[b1] Vogelstein B. & Kinzler K. W. Cancer genes and the pathways they control. Nat. Med. 10, 789–799 (2004). 15286780 10.1038/nm1087

[b2] Lo Sasso G., Petruzzelli M. & Moschetta A. A translational view on the biliary lipid secretory network. Biochim. Biophys. Acta 1781, 79–96 (2008). 18194677 10.1016/j.bbalip.2007.12.002

[b3] Meddings J. B., DeSouza D., Goel M. & Thiesen S. Glucose transport and microvillus membrane physical properties along the crypt-villus axis of the rabbit. J. Clin. Invest 85, 1099–1107 (1990). 2318967 10.1172/JCI114541 PMC296540

[b4] Shakir K. M., Sundaram S. G. & Margolis S. Lipid synthesis in isolated intestinal cells. J. Lipid Res. 19, 433–442 (1978). 659984

[b5] Dobrzynska I., Szachowicz-Petelska B., Sulkowski S. & Figaszewski Z. Changes in electric charge and phospholipids composition in human colorectal cancer cells. Mol. Cell Biochem. 276, 113–119 (2005). 16132692 10.1007/s11010-005-3557-3

[b6] Dueck D. A. . The modulation of choline phosphoglyceride metabolism in human colon cancer. Mol. Cell Biochem. 162, 97–103 (1996). 8905631 10.1007/BF00227535

[b7] Sakai K. . Composition and turnover of phospholipids and neutral lipids in human breast cancer and reference tissues. Carcinogenesis 13, 579–584 (1992). 1576710 10.1093/carcin/13.4.579

[b8] Preetha A., Banerjee R. & Huilgol N. Surface activity, lipid profiles and their implications in cervical cancer. J. Cancer Res. Ther. 1, 180–186 (2005). 17998650 10.4103/0973-1482.19600

[b9] Bussolino D. F. . c-Fos associates with the endoplasmic reticulum and activates phospholipid metabolism. FASEB J. 15, 556–558 (2001). 11259365 10.1096/fj.00-0446fje

[b10] Mori N. . Loss of p53 function in colon cancer cells results in increased phosphocholine and total choline. Mol. Imaging 3, 319–323 (2004). 15802048 10.1162/15353500200404121

[b11] Lee J. M. . A nuclear-receptor-dependent phosphatidylcholine pathway with antidiabetic effects. Nature 474, 506–510 (2011). 21614002 10.1038/nature10111 PMC3150801

[b12] Goodwin B. . A regulatory cascade of the nuclear receptors FXR, SHP-1, and LRH-1 represses bile acid biosynthesis. Mol. Cell 6, 517–526 (2000). 11030332 10.1016/s1097-2765(00)00051-4

[b13] Lee Y. K. . Liver receptor homolog-1 regulates bile acid homeostasis but is not essential for feedback regulation of bile acid synthesis. Mol. Endocrinol. 22, 1345–1356 (2008). 18323469 10.1210/me.2007-0565 PMC2409274

[b14] Mataki C. . Compromised intestinal lipid absorption in mice with a liver-specific deficiency of liver receptor homolog 1. Mol. Cell Biol. 27, 8330–8339 (2007). 17908794 10.1128/MCB.00852-07 PMC2169191

[b15] Schoonjans K. . Liver receptor homolog 1 contributes to intestinal tumor formation through effects on cell cycle and inflammation. Proc. Natl. Acad. Sci. USA 102, 2058–2062 (2005). 15684064 10.1073/pnas.0409756102 PMC548586

[b16] Cotton P. B. Non-dietary lipid in the intestinal lumen. Gut 13, 675–681 (1972). 4639402 10.1136/gut.13.9.675 PMC1412375

[b17] Smit J. J. . Homozygous disruption of the murine mdr2 P-glycoprotein gene leads to a complete absence of phospholipid from bile and to liver disease. Cell 75, 451–462 (1993). 8106172 10.1016/0092-8674(93)90380-9

[b18] Modica S. . The Intestinal Nuclear Receptor Signature With Epithelial Localization Patterns and Expression Modulation in Tumors. Gastroenterology (2009). 10.1053/j.gastro.2009.09.060 19818784

[b19] Botrugno O. A. . Synergy between LRH-1 and beta-catenin induces G1 cyclin-mediated cell proliferation. Mol. Cell 15, 499–509 (2004). 15327767 10.1016/j.molcel.2004.07.009

[b20] Ortlund E. A. . Modulation of human nuclear receptor LRH-1 activity by phospholipids and SHP. Nat. Struct. Mol. Biol. 12, 357–363 (2005). 15723037 10.1038/nsmb910

[b21] Krylova I. N. . Structural analyses reveal phosphatidyl inositols as ligands for the NR5 orphan receptors SF-1 and LRH-1. Cell 120, 343–355 (2005). 15707893 10.1016/j.cell.2005.01.024

[b22] Li Y. . Crystallographic identification and functional characterization of phospholipids as ligands for the orphan nuclear receptor steroidogenic factor-1. Mol. Cell 17, 491–502 (2005). 15721253 10.1016/j.molcel.2005.02.002

[b23] Musille P. M. . Antidiabetic phospholipid-nuclear receptor complex reveals the mechanism for phospholipid-driven gene regulation. Nat. Struct. Mol. Biol. 19, 532 (2012). 22504882 10.1038/nsmb.2279 PMC3960984

[b24] Kinzler K. W. . Identification of FAP locus genes from chromosome 5q21. Science 253, 661–665 (1991). 1651562 10.1126/science.1651562

[b25] Su L. K. . Multiple intestinal neoplasia caused by a mutation in the murine homolog of the APC gene. Science 256, 668–670 (1992). 1350108 10.1126/science.1350108

[b26] Chin J. E., Soffir R., Noonan K. E., Choi K. & Roninson I. B. Structure and expression of the human MDR (P-glycoprotein) gene family. Mol. Cell Biol. 9, 3808–3820 (1989). 2571078 10.1128/mcb.9.9.3808-3820.1989 PMC362442

[b27] Cui Y. J., Cheng X., Weaver Y. M. & Klaassen C. D. Tissue distribution, gender-divergent expression, ontogeny, and chemical induction of multidrug resistance transporter genes (Mdr1a, Mdr1b, Mdr2) in mice. Drug Metab Dispos. 37, 203–210 (2009). 18854377 10.1124/dmd.108.023721 PMC2683659

[b28] Baghdasaryan A. . Role of hepatic phospholipids in development of liver injury in Mdr2 (Abcb4) knockout mice. Liver Int. 28, 948–958 (2008). 18410282 10.1111/j.1478-3231.2008.01758.x

[b29] Coste A. . LRH-1-mediated glucocorticoid synthesis in enterocytes protects against inflammatory bowel disease. Proc. Natl. Acad. Sci. USA 104, 13098–13103 (2007). 17670946 10.1073/pnas.0702440104 PMC1941823

[b30] Lee J., Lee Y. & Moore D. D. Therapeutic Applications of LRH-1 Agonists. EMBO Conference on Nuclear Receptors. (2019).

[b31] Hwang H. C. & Clurman B. E. Cyclin E in normal and neoplastic cell cycles. Oncogene 24, 2776–2786 (2005). 15838514 10.1038/sj.onc.1208613

[b32] Donnellan R. & Chetty R. Cyclin D1 and human neoplasia. Mol. Pathol. 51, 1–7 (1998). 9624412 10.1136/mp.51.1.1 PMC395600

[b33] Merle P. . Oncogenic role of the frizzled-7/beta-catenin pathway in hepatocellular carcinoma. J. Hepatol. 43, 854–862 (2005). 16098625 10.1016/j.jhep.2005.05.018

[b34] Eckhardt E. R., Wang D. Q., Donovan J. M. & Carey M. C. Dietary sphingomyelin suppresses intestinal cholesterol absorption by decreasing thermodynamic activity of cholesterol monomers. Gastroenterology 122, 948–956 (2002). 11910347 10.1053/gast.2002.32539

[b35] Petruzzelli M. . Micellar lipid composition profoundly affects LXR-dependent cholesterol transport across CaCo2 cells. FEBS Lett. 583, 1274–1280 (2009). 19303409 10.1016/j.febslet.2009.03.021

[b36] Weisburger J. H., Wynder E. L. & Horn C. L. Nutritional factors and etiologic mechanisms in the causation of gastrointestinal cancers. Cancer 50, 2541–2549 (1982). 7139548

[b37] Willett W. C. Diet and cancer: one view at the start of the millennium. Cancer Epidemiol Biomarkers Prev 2001, 10, 3–8 (2007). 11205486

[b38] Wasan H. S., Novelli M., Bee J. & Bodmer W. F. Dietary fat influences on polyp phenotype in multiple intestinal neoplasia mice. Proc. Natl. Acad. Sci. USA 94, 3308–3313 (1997). 9096389 10.1073/pnas.94.7.3308 PMC20365

[b39] Broitman S. A. Cholesterol excretion and colon cancer. Cancer Res. 41, 3738–3740 (1981). 7260937

[b40] Cruse J. P., Lewin M. R., Ferulano G. P. & Clark C. G. Co-carcinogenic effects of dietary cholesterol in experimental colon cancer. Nature 276, 822–825 (1978). 723955 10.1038/276822a0

[b41] Voshol P. J. . Reduced plasma cholesterol and increased fecal sterol loss in multidrug resistance gene 2 P-glycoprotein-deficient mice. Gastroenterology 114, 1024–1034 (1998). 9558293 10.1016/s0016-5085(98)70323-3

[b42] Lin S. . The absence of LPA2 attenuates tumor formation in an experimental model of colitis-associated cancer. Gastroenterology 136, 1711–1720 (2009). 19328876 10.1053/j.gastro.2009.01.002 PMC2691721

[b43] Neufert C., Becker C. & Neurath M. F. An inducible mouse model of colon carcinogenesis for the analysis of sporadic and inflammation-driven tumor progression. Nat. Protoc. 2, 1998–2004 (2007). 17703211 10.1038/nprot.2007.279

