# Supplementary material for: Biliary Phospholipids Sustain Enterocyte Proliferation and Intestinal Tumor Progression via Nuclear Receptor Lrh1 in mice
Source: Sci Rep. 2016 Dec 20;6:39278. doi: 10.1038/srep39278 (PMC5171812; doi:10.1038/srep39278)
Supplement: Supplementary Information [file srep39278-s1.pdf]

Biliary Phospholipids Sustain Enterocyte Proliferation and Intestinal Tumor Progression via Nuclear Receptor Lrh1 in mice

Short Title: Biliary Phospholipids and Intestinal Tumorigenesis.

Michele Petruzzelli[1,2∫], Elena Piccinin[1,3,4∫], Claudio Pinto[2], Claudia Peres [1,4], Elena Bellafante[2], Antonio Moschetta[1,3*].

[1]*Department of Interdisciplinary Medicine, "Aldo Moro" University of Bari, 70124 Bari, Italy;*

[2]*Fondazione Mario Negri Sud, Santa Maria Imbaro, 66030 Chieti, Italy;*

[3]*National Cancer Research Center, IRCCS Istituto Oncologico "Giovanni Paolo II", 70124 Bari, Italy*

[4] *INBB, National Institute for Biostuctures and Biosystems, 00136 Rome, Italy*

Supplementary Figures Legend

Supplementary Figure 1.

(A) Immunoblots were performed with specific antibodies against PCNA, CyclinD1 and β-Actin on colon samples isolated from *Apc*$^{Min/+}$*/Abcb4*$^{+/+}$, *Apc*$^{Min/+}$*/Abcb4*$^{+/-}$ *and Apc*$^{Min/+}$*/Abcb4*$^{-/-}$. (B) Protein level of PCNA and CyclinD1were quantified and normalized against β-Actin. (C) Immunoblots on protein isolated from colon samples of iLrh1$^{+/+}$/Abcb4$^{-/-}$ and iLrh1$^{-/-}$/Abcb4$^{-/-}$ were performed using specific antibodies against CyclinD1 and β-Actin. (D) The protein level of Cyclin D1 were normalized on β-Actin.

Supplementary Figure 2.

Scheme of the crypt-to-epithelial colonic mucosal axis used for the BrdU assay reported in the results.

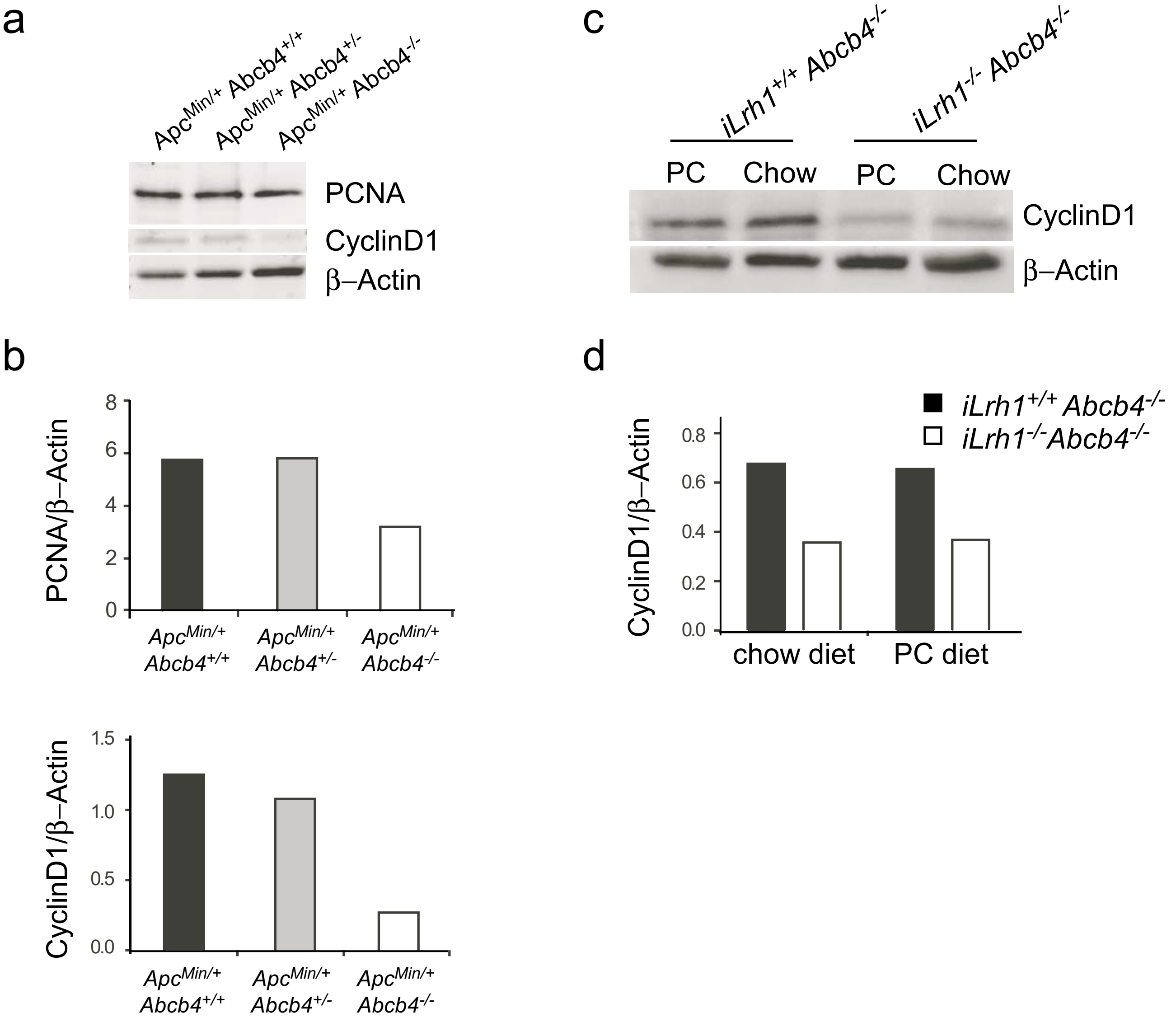

a
ApcMin/+ Abcb4+/+
ApcMin/+ Abcb4+/-
ApcMin/+ Abcb4-/-
PCNA
CyclinD1
β–Actin
b
PCNA/β–Actin
8
6
4
2
0
ApcMin/+ Abcb4+/+
ApcMin/+ Abcb4+/-
ApcMin/+ Abcb4-/-
CyclinD1/β–Actin
1.5
1.0
0.5
0.0
ApcMin/+ Abcb4+/+
ApcMin/+ Abcb4+/-
ApcMin/+ Abcb4-/-
c
iLrh1+/+ Abcb4-/-
iLrh1-/- Abcb4-/-
PC
Chow
PC
Chow
CyclinD1
β–Actin
d
iLrh1+/+ Abcb4-/-
iLrh1-/-Abcb4-/-
CyclinD1/β–Actin
0.8
0.6
0.4
0.2
0.0
chow diet
PC diet

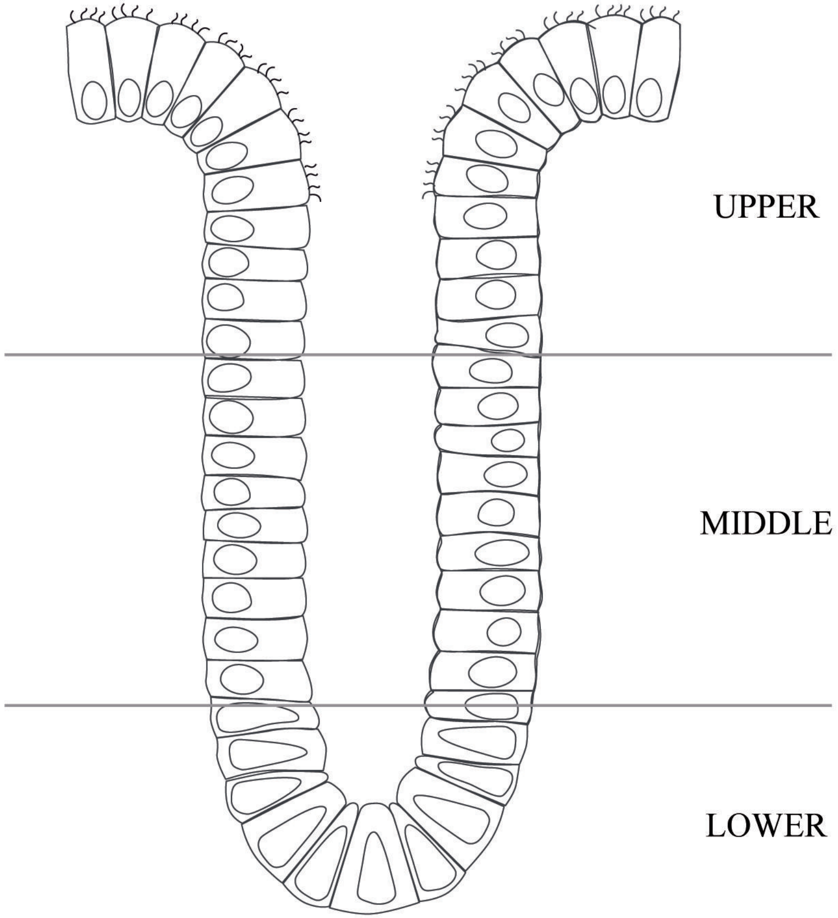
UPPER
MIDDLE
LOWER
